# Non-pharmacological intervention for neonatal pain control

**DOI:** 10.1186/1824-7288-40-S2-A52

**Published:** 2014-10-09

**Authors:** Paola Lago, Elisabetta Garetti, Anna Pirelli, Daniele Merazzi, Carlo V Bellieni, Patrizia Savant Levet, Luisa Pieragostini, Gina Ancora

**Affiliations:** 1 Woman’s and Child’s Health Department, Azienda Ospedaliera-University of Padova, Padova, Italy; 2 Dept of Pediatrics, Azienda Ospedaliero-Universitaria-Policlinico di Modena, Italy; 3 San Gerardo Hospital, Monza, Italy; 4 Dept of Women's and Children's Health, Valduce Hospital, Como, Italy; 5 Dept of Pediatrics, University Hospital, Siena, Italy; 6 Mother’s and Child’s Health Department, Maria Vittoria Hospital, Torino, Italy; 7 San Filippo Neri Hospital, Roma, Italy; 8 Azienda Ospedaliera Rimini, Italy

## Background

Acute pain and distress during medical procedures are commonplace in newborn admitted to Intensive Care Unit and can have detrimental effects, if uncontrolled.

Accumulating evidence suggests that neonate, as older children, could benefice of non pharmacological interventions (NPIs) to relive mild to moderate pain, anxiety and discomfort from minor invasive procedures. [1] These therapies include nonnutritive sucking (NNS) both with and without sucrose, swaddling, positioning, facilitated tucking (FT), kangaroo care or skin to skin contact (KMC), multi-sensorial stimulation (SS) and music therapy.

## Material and methods

To assess efficacy of NPIs for acute procedural pain in neonate, a literature search covered the period 2000-2014 via Medline and Cochrane Library database, was undertaken. Inclusion criteria were preterm and newborn, involved in randomized controlled or crossover trial. Pain reactivity was described in term of physiological parameters (heart rate, oxygen saturation) behavioral indicators (duration of first cry and total crying time) and validated unidimensional, multidimensional and/or composite pain scores as PIPP, NIPS, DAN, NFCS etc. Two independent reviewers extracted data and methodological quality was assessed, according with GRADE system.

## Results

Nineteen Randomized Controlled Trials and twelve meta-analysis and systematic reviews were taken in consideration. The efficacy of NPIs in reliving pain and distress from skin-breaking procedures has been demonstrated mostly in heel prick and venipuncture. (Table 1)

**Table 1:** Efficacy of environmental, behavioral and non-pharmacological strategies on pain reactivity in newborn.

Behavioral, cognitive and contextual interventions	Level of evidence			Grade of Recommendation
	Heel Prick	Venipuncture	Other	

**Non-nutritive sucking (NNS):** placing a pacifier or non-lactating nipple in an infant’s mouth to promote sucking behavior with no breast or formula milk to provide nourishment.	1	1	-	Strong

**Facilitated tucking:** holding the arms and legs in a flexed position	1	1	3 ET Suctioning	Strong

**Swaddling:** wrapping securely the neonate in a sheet/blanket	1	1	-	Strong

**Positioning:** laying the neonate supine	3	3	-	Weak

**Maternal touching and holding:** cradling the baby in the mother’s arms	3	3	-	Weak

**Environmental care**: controlling/ reducing light and noise, clustering care etc.	3	3	-	Weak

**Individualized developmental care** e.g. limiting environmental stimuli, lateral positioning, using supportive bedding, monitoring behavioural clues, respecting circadian rhythms	-	-	3 ROP screening	Weak

**Skin to skin or Kangaroo Mother Care** an infants is placed on their care-giver’s bare chest during a painful procedure or for soothing after a painful procedure	1	2	2 IM	Strong

**Sensorial saturation:** multiple sensorial stimulation at gustatory, auditory, olfactory and tactile level	1	-	-	Strong

**Music therapy:** music with intrauterine sounds or instrumental music in association with NNS	3	3	-	Weak

**Sucrose 24%:** in dose of 0.1-0.3 ml orally 2 minutes before the procedure in preterm infants and 1-2 ml in term infants.	1	1	-	Strong

**Breastfeeding or expressed human milk**	1	1	-	Strong

**Glucose solutions 20-30%** in dose of 1-2ml orally 2 minutes before the procedure.	1	1	-	Strong

ET suctioning= endotracheal suctioning, ROP= retinopathy of prematurity IM= intramuscular injection

Legend

1. Sufficient evidence supports efficacy for reducing pain-related behaviors (support of two or more trials)

2. Limited evidence suggests efficacy for reducing pain-related behaviors (e.g. support of 1 trial or heterogeneity among trial)

3. Limited evidence suggests inefficacy for reducing pain-related behaviors (e.g. support of 1 trial or heterogeneity among trial)

4. Sufficient evidence supports inefficacy for reducing pain-related behaviors ( support of two or more trial)

There are sufficient evidence that supports efficacy in reducing pain-relating behaviors for NNS, swaddling and FT in preterm and term neonates. [1] KMC appears to be effective, as measured by composite pain score including physiological and behavioral indicators and safe for single painful procedures, alone or combined with other NPIs. [2] Small volumes of 24% sucrose with or without NNS reduced efficiently behavioral expressions of pain and crying time, as well as PIPP scores. [3] Also expressed human milk or breastfeeding, if available, should be used to alleviate procedural pain [4], as well as 20-30% glucose [5]. SS is more effective than glucose and sucking, but there are no studies comparing SS and standard sucrose 24% and NNS with pacifier, which actually is the standard of care for heel lance. [6]

Limited evidence suggests that Music Therapy may be beneficial primarily for measures of behavior and pain, however the heterogeneity of the study preclude definitive conclusions. [7]

## Conclusions

As the efficacy of the majority of NPIs is clearly demonstrated in preterm and neonates, they should be considered for inclusion in a graduated multidisciplinary algorithm for neonatal pain management.

## References

[B1] Pillar Riddell RR Racine NM Turcotte K Uman LS Horton RE Din Osmun L Ahola Kohut S Hillgrove Stuart J Stevens B Gerwitz-Sten A Non-pharmacological management of infant and young child procedural pain Cochrane Database of Systematic Reviews 2011 10 CD006275 21975752 10.1002/14651858.CD006275.pub2

[B2] Johnston C Campbell-Yeo M Fernandes A Inglis D Streiner D Zee R Skin-to-skin for procedural pain in neonates Cochrane Database of Systematic Reviews 2014 1 CD008435 24459000 10.1002/14651858.CD008435.pub2

[B3] Stevens B Yamada J Lee GY Ohlsson A Sucrose for analgesia in newborn infants undergoing painful procedures Cochrane Database of Systematic Reviews 2013 1 CD001069 23440783 10.1002/14651858.CD001069.pub4

[B4] Shah PS Herbozo C Aliwalas LL Shah VS Breastfeeding or breast milk for procedural pain in neonates Cochrane Database of Systematic Reviews 2013 12 CD004950 10.1002/14651858.CD004950.pub3 PMC10108374 23235618

[B5] Bueno M Yamada J Harrison D Khan S Ohlsson A Adams-Webber T Beyene J Stevens B A systematic review and meta-analysis of nonsucrose sweet solutions for pain relief in neonates Pain Res Manag 2013 18 153 161 23748256 10.1155/2013/956549 PMC3673933

[B6] Bellieni CV Bagnoli F Perrone S Nenci A Cordelli DM Fusi M Ceccarelli s Buonocore G Effect of multisensory stimulation on analgesia in term neonates: a randomized controlled trial Pediatr Res 2002 51 460 3 10.1203/00006450-200204000-00010 11919330

[B7] Harling L Shaik MS Tjosvold L Leich R Liang Y Kumar M Music for medical indications in the neonatal period: a systematic review of randomized controlled trials Arch Dis Child Fetal Neonatal Ed 2009 94 F349 354 10.1136/adc.2008.148411 19477913

